# Pan-Cancer Gene Expression Predicts Per-Cancer Radiation Therapy Response

**DOI:** 10.1016/j.adro.2026.102140

**Published:** 2026-07-27

**Authors:** Harry Glickman, Shirin A. Enger

**Affiliations:** a Medical Physics Unit, Department of Oncology, Faculty of Medicine, McGill University, Montreal, Quebec, Canada; b Lady Davis Institute for Medical Research, Montreal, Quebec, Canada; c Montreal Institute for Learning Algorithms - Quebec AI Institute, Montreal, Quebec, Canada

## Abstract

**Purpose:**

Although radiation therapy response involves biological processes shared across cancers, most biomarker studies rely on small, disease-specific sample sizes that limit the reliability of prognostic models. This study evaluated whether pan-cancer data can increase the information available for estimating genomic associations with progression after radiation therapy, based on Molecular Signatures Database hallmark gene sets applied to The Cancer Genome Atlas patients.

**Methods and Materials:**

Cross-cancer predictive potential was evaluated in cancer types with at least 100 patients who received radiation therapy and had information on gene expression, progression, age, stage, and gender: breast invasive carcinoma (n = 553), cervical squamous cell carcinoma (n = 183), head-neck squamous cell carcinoma (n = 320), low-grade glioma (n = 316), lung adenocarcinoma (n = 104), skin cutaneous melanoma (n = 125), thyroid cancer (n = 326), and uterine corpus endometrial carcinoma (n=257). Gene set expression scores were calculated at the single-sample level. For each evaluation cancer type, scores from all other types were used to fit pan-cancer Cox proportional hazards models. Linear predictions from these models were evaluated in per-cancer prognostication via Kaplan–Meier curves, Cox proportional hazards estimates, including adjustment for age, stage, and gender, and optimism-corrected C-statistic and Nagelkerke pseudo-R^2^.

**Results:**

In Kaplan–Meier analysis, pan-cancer scores predicted progression in all cancer types, with varying statistical significance. Univariable Cox proportional hazards models also demonstrated consistent association with progression, again with varying significance. Associations persisted when adjusted for clinical variables, and model performance metrics indicated the potential to improve per-cancer prognostication. Associations were particularly strong for breast invasive carcinoma, cervical squamous cell carcinoma, head-neck squamous cell carcinoma, low-grade glioma, and lung adenocarcinoma, with statistically insignificant results for skin cutaneous melanoma, thyroid cancer, and uterine corpus endometrial carcinoma.

**Conclusions:**

Pan-cancer gene expression data can predict per-cancer progression after radiation therapy, with variable strength across cancer types. These findings support the use of cross-cancer genomic information to address sample size limitations that affect radiation therapy biomarker development.

## Introduction

Radiation therapy is generally prescribed using a “one size fits all” approach, a substantial gap in potential effectiveness.^1^^,^^2^ Radiation therapy response depends on biological processes that are present across many cancer types, including DNA damage response, cell cycle control, hypoxia, and immune signaling.^2^, ^3^, ^4^, ^5^, ^6^ This commonality has been leveraged by the genomic-adjusted radiation dose studies to predict survival across several cancer types.^7^^,^^8^ Despite this, most genomic prediction studies in radiation therapy are developed within single disease sites.^9^, ^10^, ^11^, ^12^, ^13^ This limits sample size and the number of outcome events available for model development, which reduces the stability and reproducibility of prognostic estimates.^14^, ^15^, ^16^, ^17^ This is especially problematic in genomic studies, where there are thousands of potential predictor parameters,^18^ and with the increasing use of modern machine learning methods, which require a higher sample size to ensure reliable and generalizable results.^19^ Indeed, while sample sizes of 10-20 events per predictor parameter (EPP) are typically appropriate for traditional multivariable statistical models, more than 200 EPP may be necessary for modern machine learning techniques.^19^^,^^20^

Increasing sample size by pooling data across institutions is often difficult because of regulatory, governance, and data harmonization barriers. An alternative approach is to use genomic information from multiple cancer types to improve estimation while evaluating performance within the target cancer type. This study examined whether pan-cancer gene expression information can improve prediction of progression after radiation therapy under limited sample size. It leveraged The Cancer Genome Atlas (TCGA) database, containing standardized genomic and clinical data for over 11,000 patients, from multiple clinical centers and for over 30 cancer types.^21^ Gene set analysis was used to investigate overlapping biological functions of genes, based on the Molecular Signature Database hallmark gene set collection.^22^

## Methods and Materials

Gene expression data (transcripts per million) for all primary tumor samples in the TCGA were acquired from the National Cancer Institute Genomic Data Commons.^23^ Single-sample scoring of molecular phenotypes (singscore)^24^ was applied to each patient for all 50 Molecular Signature Database hallmark gene sets using the singscore R package. Two metrics were calculated for each gene set: the “score” (mean percentile gene rank), and the “dispersion” (median absolute deviation of gene ranks). When multiple samples were available for a single patient, scores and dispersions were averaged across samples. This produced 100 separate genomic parameters for each patient. All statistical analyses were conducted in R version 4.4.1, using tidyverse R packages for data manipulation and visualization. We provide the full analysis code at https://github.com/HarryGlickman/Engerlab_Pan_Cancer_GeneSets.

Age, gender, stage, cancer type, and treatment information were acquired from TCGA clinical data files. All variants of unknown or missing data (eg, [Not Available], [Not Applicable], [Unknown]) were considered missing. Age was treated as a linear variable. Gender was treated as a binary variable and used as a proxy for sex. Tumor stage was grouped into 4 categories (I, II, III, and IV) by merging all stage subcategories (eg, IA, IB2, and IC). Pathological stage was used where available; otherwise, clinical stage was used. For low-grade glioma, histological grade was used instead of stage as these cancers are not staged. Cancer type was treated as a categorical variable. Patients who had a “Radiation Therapy, NOS” value of “yes” were considered to have received radiation therapy. Progression-free interval (PFI) was the outcome event, drawn from Liu et al.'s TCGA pan-cancer outcome data.^25^ PFI is measured from the date of diagnosis to the date of a new tumor event, including disease progression, locoregional recurrence, distant metastasis, a new primary tumor, or death with tumor.^25^ For the TCGA-LAML project, PFI was not available, so overall survival was used as the outcome variable.

Cancer types with over 100 patients who received radiation therapy and had complete information on age, gender, stage, and gene expression were used for evaluation. Nonevaluation cancer types were grouped together in an *OTHER* type to reduce parameters. One evaluation type at a time, all other types were used to fit a pan-cancer Cox proportional hazards (CoxPH) model using cancer type and all 100 genomic parameters as predictors. Patients were included in this pan-cancer genomic model if they had gene expression, cancer type, and outcome data available. Linear predictions were then extracted for each evaluation case. The association of these predictions with per-cancer outcomes was assessed by splitting predictions at the median and constructing Kaplan–Meier curves with log-rank tests using the *survminer* and ggsurvfit R packages, and by fitting univariable per-cancer CoxPH models and extracting coefficients, using the survival R package. Whether these associations persisted when adjusted for clinical variables was assessed through the use of multivariable CoxPH models including age, stage, and gender. Per-cancer genomic scores were also generated for comparison using an out-of-fold validation framework, described and presented in the supplementary materials.

The performance of per-cancer CoxPH models was assessed via optimism-corrected C-statistic and Nagelkerke $R^{2}$, treating pan-cancer predictions as fixed variables in per-cancer validations. Bootstraps were generated, and optimism correction applied using the validate function from the rms R package with 500 bootstraps, with 95% CIs calculated using Harrell’s asymmetric bootstrap confidence limits for overfitting-corrected model performance method. CoxPH model assumptions of pan-cancer and per-cancer models were tested statistically and graphically, described in the supplementary materials.

## Results

Of 11,080 patients in the TCGA clinical data files, 3269 received confirmed radiation therapy and had complete information on gene expression and outcome. Among these, 8 cancer types had over 100 patients with complete data on age, stage, and gender: breast invasive carcinoma (BRCA, n=553), cervical squamous cell carcinoma (CESC, n=183), head-neck squamous cell carcinoma (HNSC,n=320), low-grade glioma (LGG,n=316), lung adenocarcinoma (LUAD, n=104), skin cutaneous melanoma (SKCM, n=125), thyroid cancer (n = 326), and uterine corpus endometrial carcinoma (n = 257). Table 1 presents sample size, progression status, follow-up time, degrees of freedom for prediction at 10 to 20 EPP, complete cases, age, stage, and gender for these 8 evaluation types, for nonevaluation types, and for all pan-cancer patients who received confirmed radiation therapy.

**Table 1:** Patient characteristics

Cancer type	Cases	Progression (PFI)		Follow-up days mean (SD)	Degrees of freedom (20-10 EPP)	Complete cases (age, stage, gender)	Age mean (SD)	Stage				Gender	
		1	0					1	2	3	4	M	F
BRCA	565	82	483	1265 (1053.7)	4-8	553	56.84 (12.22)	92	288	165	8	4	561
CESC	187	58	129	1123.46 (1172.04)	3-6	183	48.73 (14.04)	84	51	31	17	0	187
HNSC	320	129	191	818.96 (799.69)	6-13	320	59.54 (10.96)	12	28	52	228	249	71
LGG	317	146	171	787.52 (733.72)	7-15	316	44.24 (13.53)	0	108	208	0	176	141
LUAD	107	89	18	586.07 (510.77)	4-9	104	64.49 (10.91)	42	24	31	9	42	65
SKCM	139	125	14	1395.67 (1613.8)	6-12	125	54.86 (16.17)	38	23	60	4	94	45
THCA	327	47	280	1224.58 (1011.3)	2-5	326	46.89 (15.76)	166	31	83	46	101	226
UCEC	258	75	183	1053.19 (879.59)	4-8	257	63.44 (10.29)	149	34	64	11	0	258
OTHER	1049	696	353	661.99 (784.82)	35-70	613	59.41 (12.78)	93	229	206	88	690	359
ALL	3269	1447	1822	936.01 (966.34)	72-145	2797	55.92 (14.26)	676	816	900	411	1356	1913

*Abbreviations*: BRCA = breast invasive carcinoma; CESC = cervical squamous cell carcinoma; HNSC = head-neck squamous cell carcinoma; LGG = low-grade glioma; LUAD = lung adenocarcinoma; PFI = progression-free interval; SKCM = skin cutaneous melanoma; THCA = thyroid cancer; UCEC = uterine corpus endometrial carcinoma; M = male; F = female.

Sample size, progression status, follow-up time, degrees of freedom for prediction at 10 to 20 events per parameter (EPP), complete cases, age, stage, and gender stratified by cancer type for all patients who received confirmed radiation therapy and who had gene expression data available. For LGG, staging information is replaced by histological grade. OTHER refers to nonevaluation cancer types. ALL refers to all cancer types grouped together.

Figure 1 presents averaged and standardized single-sample gene set scores and dispersions for the 8 evaluation types, for nonevaluation types, and for all pan-cancer data, stratified by progression status. Gene set parameters are organized by the largest pan-cancer differential between those who progressed and those who did not.

**Figure 1:**
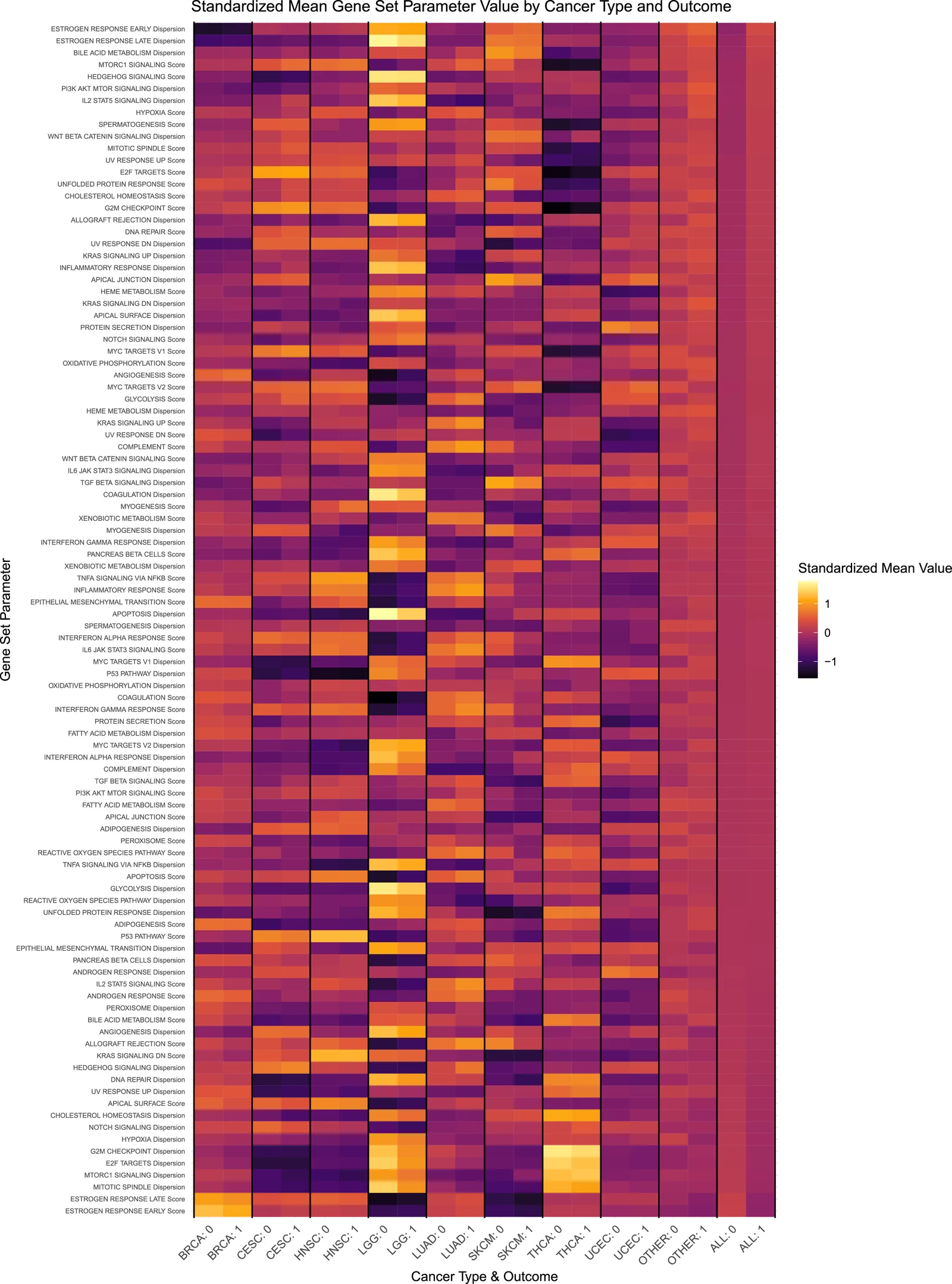
Gene set parameters. Averaged 100 single-sample gene set scores and dispersions presented for the 8 evaluation types, for nonevaluation types (OTHER), and for all pan-cancer data (ALL), stratified by progression status (PFI). Gene set parameters are standardized across all patients, and organized by the largest pan-cancer differential between those who progressed and those who did not. *Abbreviations*: BRCA = breast invasive carcinoma; CESC = cervical squamous cell carcinoma; HNSC = head-neck squamous cell carcinoma; LGG = low-grade glioma; LUAD = lung adenocarcinoma; SKCM = skin cutaneous melanoma; THCA = thyroid cancer; UCEC = uterine corpus endometrial carcinoma.

For all 8 evaluation cancer types separately, a different pan-cancer CoxPH model was fit using all 100 gene set parameters and cancer type, for all cases without the cancer type of interest. For reporting, a single pan-cancer model was created without withholding any types, and by standardizing and centering all variables for interpretation. Pan-cancer hazard ratios, CIs, and *P* values for the 100 gene parameters and 8 type parameters, with no cancer types withheld, are presented in Table 2. Variance inflation factors are large, ranging from 2.3 to 153.6, with a median of 9.4 and an IQR of 5.2 to 17.0.

**Table 2:** Pan-cancer CoxPH coefficients

Variable	HR [95% CI] *P* value	Variable	HR [95% CI] *P* value
TNFA SIGNALING VIA NFKB score	0.61 [0.43−0.86] *P* = .004	MYC TARGETS V1 score	0.92 [0.64−1.32] *P* = .643
TNFA SIGNALING VIA NFKB dispersion	0.73 [0.59−0.91] *P* = .005	MYC TARGETS V1 dispersion	1.14 [0.95−1.38] *P* = .156
HYPOXIA score	0.97 [0.78−1.2] *P* = .770	MYC TARGETS V2 score	0.89 [0.72−1.1] *P* = .282
HYPOXIA dispersion	1 [0.88−1.15] *P* = .950	MYC TARGETS V2 dispersion	0.97 [0.87−1.08] *P* = .560
CHOLESTEROL HOMEOSTASIS score	0.88 [0.77−1.01] *P* = .073	EPITHELIAL MESENCHYMAL TRANSITION score	1.61 [1.16−2.23] *P* = .005
CHOLESTEROL HOMEOSTASIS dispersion	0.94 [0.84−1.04] *P* = .222	EPITHELIAL MESENCHYMAL TRANSITION dispersion	1.44 [1.14−1.81] *P* = .002
MITOTIC SPINDLE score	0.73 [0.53−1.01] *P* = .054	INFLAMMATORY RESPONSE score	1.72 [1.19−2.5] *P* = .004
MITOTIC SPINDLE dispersion	0.96 [0.84−1.1] *P* = .556	INFLAMMATORY RESPONSE dispersion	1.29 [1.08−1.53] *P* = .004
WNT BETA CATENIN SIGNALING score	0.98 [0.87−1.11] *P* = .794	XENOBIOTIC METABOLISM score	0.92 [0.78−1.08] *P* = .283
WNT BETA CATENIN SIGNALING dispersion	1.08 [1−1.16] *P* = .062	XENOBIOTIC METABOLISM dispersion	0.91 [0.79−1.05] *P* = .197
TGF BETA SIGNALING score	1.26 [1.07−1.48] *P* = .005	FATTY ACID METABOLISM score	0.88 [0.77−1.01] *P* = .075
TGF BETA SIGNALING dispersion	1.1 [1−1.21] *P* = .062	FATTY ACID METABOLISM dispersion	1.03 [0.92−1.16] *P* = .575
IL6 JAK STAT3 SIGNALING score	0.95 [0.72−1.25] *P* = .701	OXIDATIVE PHOSPHORYLATION score	0.93 [0.77−1.12] *P* = .439
IL6 JAK STAT3 SIGNALING dispersion	1.07 [0.91−1.26] *P* = .439	OXIDATIVE PHOSPHORYLATION dispersion	0.98 [0.86−1.12] *P* = .785
DNA REPAIR score	0.98 [0.83−1.16] *P* = .817	GLYCOLYSIS score	1.15 [0.96−1.39] *P* = .138
DNA REPAIR dispersion	1.03 [0.92−1.16] *P* = .588	GLYCOLYSIS dispersion	1.03 [0.88−1.2] *P* = .721
G2M CHECKPOINT score	1.26 [0.63−2.52] *P* = .513	REACTIVE OXYGEN SPECIES PATHWAY score	0.99 [0.89−1.1] *P* = .859
G2M CHECKPOINT dispersion	0.81 [0.57−1.15] *P* = .237	REACTIVE OXYGEN SPECIES PATHWAY dispersion	0.96 [0.87−1.05] *P* = .386
APOPTOSIS score	1.22 [0.95−1.58] *P* = .123	P53 PATHWAY score	0.87 [0.7−1.09] *P* = .238
APOPTOSIS dispersion	1.09 [0.93−1.27] *P* = .288	P53 PATHWAY dispersion	0.88 [0.77−0.99] *P* = .039
NOTCH SIGNALING score	0.95 [0.81−1.1] *P* = .474	UV RESPONSE UP score	0.99 [0.85−1.15] *P* = .856
NOTCH SIGNALING dispersion	1.05 [0.96−1.15] *P* = .287	UV RESPONSE UP dispersion	0.95 [0.86−1.05] *P* = .355
ADIPOGENESIS score	1.27 [1.07−1.51] *P* = .006	UV RESPONSE DN score	0.79 [0.6−1.05] *P* = .104
ADIPOGENESIS dispersion	0.95 [0.85−1.06] *P* = .378	UV RESPONSE DN dispersion	1.03 [0.95−1.11] *P* = .532
ESTROGEN RESPONSE EARLY score	1.54 [1.04−2.26] *P* = .029	ANGIOGENESIS score	0.96 [0.84−1.11] *P* = .617
ESTROGEN RESPONSE EARLY dispersion	0.94 [0.77−1.14] *P* = .539	ANGIOGENESIS dispersion	0.98 [0.89−1.08] *P* = .668
ESTROGEN RESPONSE LATE score	0.46 [0.32-0.67] *P* < .001	HEME METABOLISM score	0.99 [0.86−1.13] *P* = .883
ESTROGEN RESPONSE LATE dispersion	0.98 [0.79−1.21] *P* = .866	HEME METABOLISM dispersion	0.96 [0.88−1.04] *P* = .288
ANDROGEN RESPONSE score	1.02 [0.86−1.21] *P* = .831	COAGULATION score	1.19 [1−1.41] *P* = .053
ANDROGEN RESPONSE dispersion	1.06 [0.95−1.17] *P* = .306	COAGULATION dispersion	0.86 [0.73−1.01] *P* = .065
MYOGENESIS score	0.9 [0.76−1.06] *P* = .222	IL2 STAT5 SIGNALING score	0.8 [0.58−1.1] *P* = .174
MYOGENESIS dispersion	0.89 [0.77−1.02] *P* = .093	IL2 STAT5 SIGNALING dispersion	1.02 [0.88−1.18] *P* = .815
PROTEIN SECRETION score	1.08 [0.89−1.33] *P* = .434	BILE ACID METABOLISM score	1.05 [0.9−1.24] *P* = .519
PROTEIN SECRETION dispersion	1.12 [1.01−1.25] *P* = .037	BILE ACID METABOLISM dispersion	1.12 [1.02−1.24] *P* = .022
INTERFERON ALPHA RESPONSE score	0.83 [0.54−1.27] *P* = .393	PEROXISOME score	1.04 [0.93−1.17] *P* = .474
INTERFERON ALPHA RESPONSE dispersion	0.83 [0.67−1.04] *P* = .100	PEROXISOME dispersion	0.99 [0.91−1.08] *P* = .858
INTERFERON GAMMA RESPONSE score	1.39 [0.75−2.58] *P* = .296	ALLOGRAFT REJECTION score	0.56 [0.38−0.84] *P* = .005
INTERFERON GAMMA RESPONSE dispersion	1.05 [0.85−1.28] *P* = .666	ALLOGRAFT REJECTION dispersion	0.95 [0.75−1.19] *P* = .645
APICAL JUNCTION score	0.89 [0.72−1.1] *P* = .261	SPERMATOGENESIS score	0.96 [0.85−1.09] *P* = .568
APICAL JUNCTION dispersion	0.95 [0.85−1.07] *P* = .392	SPERMATOGENESIS dispersion	1.09 [0.98−1.21] *P* = .126
APICAL SURFACE score	0.97 [0.84-1.1] *P* = .608	KRAS SIGNALING UP score	1.3 [1.03−1.63] *P* = .024
APICAL SURFACE dispersion	1.01 [0.92−1.1] *P* = .853	KRAS SIGNALING UP dispersion	0.93 [0.85−1.02] *P* = .145
HEDGEHOG SIGNALING score	1.25 [1.06−1.47] *P* = .008	KRAS SIGNALING DN score	0.97 [0.86−1.11] *P* = .684
HEDGEHOG SIGNALING dispersion	1.1 [1.01−1.19] *P* = .022	KRAS SIGNALING DN dispersion	0.97 [0.87−1.08] *P* = .530
COMPLEMENT score	1.07 [0.81−1.4] *P* = .654	PANCREAS BETA CELLS score	0.97 [0.88−1.07] *P* = .549
COMPLEMENT dispersion	1.22 [1.07−1.4] *P* = .003	PANCREAS BETA CELLS dispersion	1 [0.92−1.09] *P* = .926
UNFOLDED PROTEIN RESPONSE score	0.99 [0.81−1.2] *P* = .899	Type: CESC	3.12 [1.9−5.11] *P* < .001
UNFOLDED PROTEIN RESPONSE dispersion	1 [0.91−1.09] *P* = .945	Type: HNSC	4.8 [3−7.66] *P* < .001
PI3K AKT MTOR SIGNALING score	1.11 [0.96−1.28] *P* = .145	Type: LGG	4.93 [2.92−8.35] *P*<.001
PI3K AKT MTOR SIGNALING dispersion	1.07 [1−1.16] *P* = .067	Type: LUAD	8.36 [5.31−13.16] *P* < .001
MTORC1 SIGNALING score	1.3 [0.97−1.75] *P* = .084	Type: OTHER	7.75 [5.27−11.42] *P* < .001
MTORC1 SIGNALING dispersion	1.06 [0.9−1.24] *P* = .517	Type: SKCM	4.57 [2.83−7.37] *P* <.001
E2F TARGETS score	1.23 [0.63−2.43] *P* = .542	Type: THCA	1.02 [0.56−1.86] *P* = .941
E2F TARGETS dispersion	1 [0.7−1.42] *P* = .986	Type: UCEC	2.42 [1.53−3.84] *P* <.001

*Abbreviations*: BRCA = breast invasive carcinoma; CESC = cervical squamous cell carcinoma; HNSC = head-neck squamous cell carcinoma; LGG = low-grade glioma; LUAD = lung adenocarcinoma; PFI = progression-free interval; SKCM = skin cutaneous melanoma; THCA = thyroid cancer; UCEC = uterine corpus endometrial carcinoma.

Hazard ratios (HR), 95% CIs, and *P* values for pan-cancer parameters, without withholding any cancer types, and with variables standardized. For type parameters, BRCA is the reference category.

Pan-cancer linear predictions stratified by cancer type and outcome for each evaluation type are presented in Fig. S1. Pan-cancer predictions for each evaluation type were stratified at the median and used to construct Kaplan–Meier curves, with log-rank tests and 95% CIs, presented with risk tables in Fig. 2. Pan-cancer scores were predictive of progression in all cancer types, with varying levels of statistical significance.

**Figure 2:**
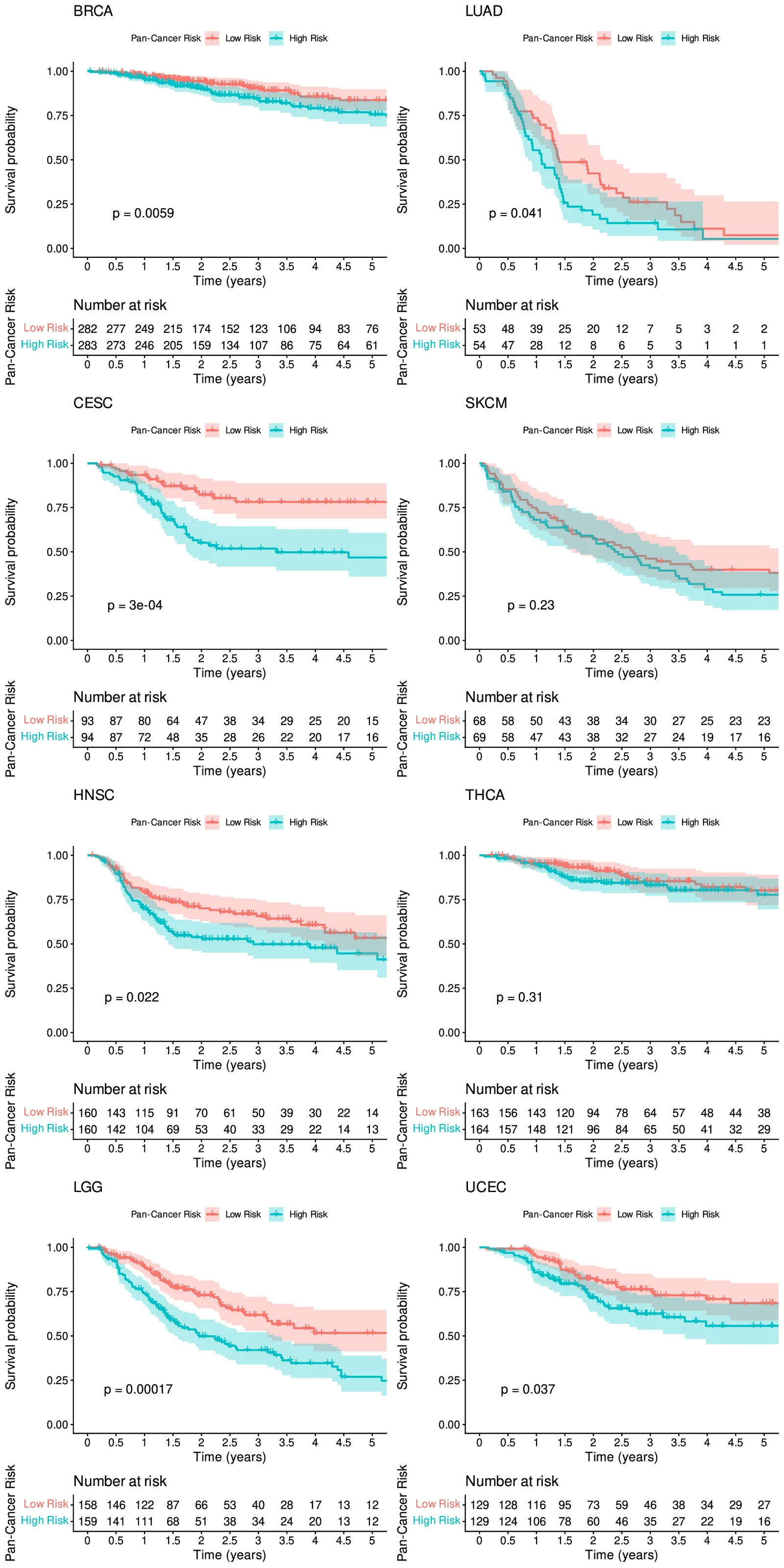
Kaplan–Meier curves. Kaplan–Meier curves for pan-cancer linear predictions stratified at the median for each evaluation cancer type, with log-rank tests and 95% CIs, presented with risk tables. *Abbreviations*: BRCA = breast invasive carcinoma; CESC = cervical squamous cell carcinoma; HNSC = head-neck squamous cell carcinoma; LGG = low-grade glioma; LUAD = lung adenocarcinoma; PFI = progression-free interval; SKCM = skin cutaneous melanoma; THCA = thyroid cancer; UCEC = uterine corpus endometrial carcinoma.

Hazard ratios, 95% CIs, and *P* values for univariable and multivariable CoxPH models constructed using pan-cancer predictions and basic clinical variables (age, stage, and gender) are presented in Table 3. Pan-cancer genomic scores were standardized within each cancer type. Model 1 is progression as a function of pan-cancer predictions, for each cancer type. Model 2 is progression as a function of pan-cancer predictions and clinical variables, for each cancer type. Pan-cancer scores were associated with progression in all cancer types, with varying levels of statistical significance. CoxPH coefficients for out-of-fold per-cancer predictions are presented in Table S1. Using all 100 genomic parameters for per-cancer predictions was inaccurate for most cancer types. Dimension reduction to 10 or 20 EPP using principal component analysis was more accurate for certain cancer types, but the association of pan-cancer predictions with progression largely persisted when adjusted for these per-cancer predictions.

**Table 3:** Per-cancer CoxPH coefficients

		Model 1: Pan-Cancer Predictions						
	BRCA	CESC	HNSC	LGG	LUAD	SKCM	THCA	UCEC
Pan-cancer predictions	1.42 [1.14−1.76] *P* = .002	1.95 [1.48−2.56] *P*<.001	1.3 [1.09−1.54] *P* = .003	1.54 [1.31-1.81] *P* < .001	1.35 [1.09−1.68] *P* = .006	1.04 [0.87−1.24] *P* = .677	1.1 [0.81−1.48] *P* = .548	1.11 [0.9−1.38] *P* = .329

		Model 2: Pan-Cancer Predictions + Age + Stage (+ Gender)						
	BRCA	CESC	HNSC	LGG	LUAD	SKCM	THCA	UCEC
Pan-cancer predictions	1.37 [1.09−1.73] *P* = .007	2.05 [1.53-2.75] *P* < .001	1.29 [1.08−1.54] *P* = .005	1.49 [1.27−1.76] *P*<.001	1.47 [1.17−1.85] *P* = .001	1.08 [0.88−1.31] *P* = .462	1.04 [0.76−1.42] *P* = .804	1.08 [0.86−1.37] *P* = .504
Age	1 [0.98−1.02] *P* = .941	1 [0.99−1.02] *P* = .615	1.02 [1−1.04] *P* = .028	1.02 [1.01−1.04] *P*<.001	1.01 [0.99−1.03] *P* = .353	1.02 [1.01−1.03] *P* = .003	1.01 [0.98−1.04] *P* = .431	1 [0.98−1.03] *P* = .753
Stage 2	1.64 [0.71−3.77] *P* = .243	0.61 [0.31−1.21] *P* = .154	0.84 [0.28−2.52] *P* = .751	0.9 [0.63−1.3] *P* = .584	1.77 [1−3.14] *P* = .051	1.18 [0.66−2.1] *P* = .584	1.04 [0.3−3.58] *P* = .949	0.79 [0.35−1.8] *P* = .58
Stage 3	3.68 [1.62−8.38] *P* = .002	0.6 [0.26−1.39] *P* = .237	0.86 [0.31−2.35] *P* = .762	NA	1.39 [0.81−2.39] *P* = .227	2.44 [1.53-3.91] *P* < .001	1.54 [0.6−3.98] *P* = .369	1.62 [0.95−2.77] *P* = .078
Stage 4	22.5 [7.77−65.16] *P* <.001	1.57 [0.77−3.22] *P* = .216	1.2 [0.48−2.97] *P* = .695	NA	0.72 [0.29−1.78] *P* = .479	3.56 [1.22−10.42] *P* = .02	2.18 [0.8−5.93] *P* = .126	8.11 [3.93−16.74] *P* < .001
Male	NA	NA	0.89 [0.59−1.35] *P* = .584	0.99 [0.71−1.38] *P* = .971	0.95 [0.6−1.5] *P* = .811	1.04 [0.69−1.58] *P* = .848	0.99 [0.53−1.85] *P* = .966	NA

*Abbreviations*: BRCA = breast invasive carcinoma; CESC = cervical squamous cell carcinoma; CoxPH = Cox proportional hazards; HNSC = head-neck squamous cell carcinoma; LGG = low-grade glioma; LUAD = lung adenocarcinoma; PFI = progression-free interval; SKCM = skin cutaneous melanoma; THCA = thyroid cancer; UCEC = uterine corpus endometrial carcinoma.

Hazard ratios (HR), 95% CI, and *P* values for pan-cancer predictions with and without clinical adjustment. Pan-cancer predictions were standardized per cancer type. For stage parameters, “Stage 1” is the reference category, except for LGG for which “Stage 3” is the reference category, referring to grade for that cancer type. For gender, “Female” is the reference category. Coefficients are formatted as HR [95% CI] *P* value.

Model performance metrics were calculated for CoxPH models created using pan-cancer predictions and basic clinical variables. Bootstrap-corrected C-statistics and Nagelkerke pseudo-$R^{2}$ values are presented in Table 4. There were convergence issues in SKCM bootstrap validations, and model performance metrics may be unreliable for that cancer type.

**Table 4:** Per-cancer model performance

	C-statistic [95% CI]		
	Clinical	Pan-Cancer only	Clinical + Pan-Cancer
BRCA	0.66 [0.59−0.73]	0.57 [0.51−0.64]	0.68 [0.61−0.75]
CESC	0.53 [0.46−0.59]	0.69 [0.62−0.76]	0.69 [0.63−0.76]
HNSC	0.52 [0.46−0.57]	0.58 [0.53−0.63]	0.56 [0.51−0.6]
LGG	0.6 [0.55−0.65]	0.63 [0.58−0.68]	0.64 [0.59−0.69]
LUAD	0.54 [0.47−0.6]	0.59 [0.52−0.66]	0.6 [0.54−0.66]
SKCM	0.64 [0.59−0.7]	0.51 [0.45−0.57]	0.64 [0.59−0.69]
THCA	0.55 [0.46−0.62]	0.53 [0.44−0.6]	0.54 [0.46−0.62]
UCEC	0.6 [0.54−0.67]	0.54 [0.47−0.6]	0.61 [0.54−0.67]

	Nagelkerke pseudo-R^2^ [95% CI]		
	Clinical	Pan-Cancer only	Clinical +Pan-Cancer
BRCA	0.06 [0−0.11]	0.01 [−0.01to 0.03]	0.08 [0.02−0.13]
CESC	0.02 [−0.07 to 0.08]	0.14 [0.05-0.22]	0.13 [0.02−0.23]
HNSC	0 [−0.04 to 0.04]	0.02 [−0.02 to 0.05]	0.02 [−0.04 to 0.07]
LGG	0.04 [−0.02 to 0.08]	0.07 [0.01−0.12]	0.1 [0.03−0.16]
LUAD	−0.02 [−0.14 to 0.06]	0.07 [-0.05 to 0.17]	0.06 [−0.09 to 0.18]
SKCM	0.13 [−0.01 to 0.27]	0 [−0.05 to 0.02]	0.13 [−0.01 to 0.27]
THCA	0 [−0.06 to 0.05]	0 [−0.02 to 0.01]	0 [−0.06 to 0.04]
UCEC	0.07 [0−0.14]	0 [−0.02 to 0.01]	0.07 [−0.01 to 0.14]

*Abbreviations*: BRCA = breast invasive carcinoma; CESC = cervical squamous cell carcinoma; CoxPH = Cox proportional hazards; HNSC = head-neck squamous cell carcinoma; LGG = low-grade glioma; LUAD = lung adenocarcinoma; SKCM = skin cutaneous melanoma; THCA = thyroid cancer; UCEC = uterine corpus endometrial carcinoma.

Optimism-corrected C-statistic and Nagelkerke pseudo- *R*^2^ with 95% CI for models with clinical information only, pan-cancer genomic predictions only, or both clinical and pan-cancer predictions. A total of 500 bootstraps were used, and 95% CIs were generated using the asymmetric bootstrap confidence limits for the overfitting-corrected model performance method. There were convergence issues in some bootstraps of SKCM, and results may be unreliable for that cancer type.

## Discussion

In this study, pan-cancer genomic scores were derived using data from multiple cancer types and evaluated using a leave-one-cancer-type-out validation that excluded the target cancer type from model training. This approach allows shared genomic associations with progression after radiation therapy to be estimated while preserving cancer-specific evaluation. Single-sample gene set scores varied according to both cancer type and progression status for TCGA patients who received radiation therapy. In CoxPH models adjusted for all 100 gene set parameters and cancer type, several individual parameters were strongly related to progression, despite high variance inflation. Linear predictions were extracted from this pan-cancer model for use in per-cancer prognostication in 8 evaluation cancer types. This composite linear predictor was used to assess the combined prognostic potential of pan-cancer gene sets, rather than specific gene set estimates, which are individually difficult to interpret.

Pan-cancer linear predictions were associated with progression in all evaluation types when split at the median for Kaplan–Meier analysis, notably for BRCA, CESC, and LGG, with varying statistical significance in log-rank tests. In univariable CoxPH models, pan-cancer scores predicted progression in all evaluation types, again with varying statistical significance, with stronger results for BRCA, CESC, HNSC, LGG, and LUAD. Coefficients were highly significant for several cancer types, even when applying conservative Bonferroni correction for the 8 evaluation types, with an adjusted significance threshold of approximately 0.006. These large associations persisted when adjusted for age, stage, gender, and per-cancer predictions. For CESC, the cancer type with the most extreme results, a 1-unit increase in standardized pan-cancer score was associated with 1.95 times the rate of progressing, with 95% CIs from 1.48 to 2.56(P<.001). When CESC outcomes were modeled as a function of pan-cancer predictions, age, and stage, a 1-unit increase in standardized scores was still associated with 2.05 times the rate of progressing, with 95% CIs from 1.53 to 2.75(P<.001). A similar pattern was observed in all cancer types, with maintenance of the univariable hazard ratio and CIs when adjusting for clinical parameters.

Per-cancer CoxPH assumptions of proportional hazards, linearity, and influential observations were largely satisfied, further increasing confidence in the results. Optimism-corrected model performance metrics showed the potential to increase predictive performance for certain cancer types, especially for CESC, HNSC, LGG, and LUAD. For CESC in particular, optimism-corrected C-statistic increased by a remarkable 0.16. For BRCA, although pan-cancer scores were strongly associated with progression, model performance scores increased only marginally. For UCEC, THCA, and SKCM, there were no obvious performance improvements. All model performance metrics had large CIs due to relatively limited per-cancer sample sizes. When the same 100 gene set parameters were used to create out-of-fold per-cancer predictions, per-cancer predictions were inaccurate for most cancer types. When a more appropriate number of parameters were used, applying a typical rule of thumb of between 10 and 20 EPP, per-cancer predictions were more accurate, but adjusted pan-cancer scores largely remained associated with progression.

The study is limited by several important factors. First, regarding participant selection, radiation therapy treatment status as an inclusion criterion is problematic because treatment data are optional within the TCGA, with a high degree of missingness, which likely introduced selection bias. Second, regarding genomic scores, for all participants a single set of scores was generated, even when multiple samples were available, ignoring important intratumor genetic heterogeneity in both space and time. Also, hallmark gene sets were chosen due to their ubiquity, and singscore due to its simple single-sample properties, but results could differ for other analytic decisions and genetic parameters. Third, regarding pan-cancer modeling, CoxPH was chosen due to its common use, but more sophisticated models allowing for interactions between genomic parameters should result in better pan-cancer representations of progression following radiation therapy. Fourth, per-cancer models were generally poorly calibrated, and it is not difficult to improve relative to a poorly calibrated model. Age and tumor stage, however, are often the main factors used for oncological treatment planning and prognostication, and therefore, consistently large associations adjusted for these standard factors are likely to be clinically significant.

Interpretation of pan-cancer linear predictions was also limited. Some hallmark gene set parameters are directly related to radiation therapy response, such as reactive oxygen species or mitotic spindle pathways, while others are less directly related, like cholesterol homeostasis or spermatogenesis. Also, pan-cancer predictions were controlled for cancer type, addressing differing mean rates of cancer-specific mortality, but cancer types with larger samples still had more influence in the pan-cancer fit. Furthermore, pan-cancer predictions were not controlled for stage or other clinical factors, the inclusion of which could plausibly improve or blunt the predictive performance of the pan-cancer predictions. Adjusting for stage but excluding stage coefficients from the linear predictions, for instance, would likely decrease the per-cancer univariable associations with progression but could increase the multivariable associations and improve performance metrics by making genomic predictions more independent of stage. There were also technical issues with the pan-cancer models, including high variance inflation and violations of the proportional hazards assumptions for many covariates, including cancer type. Conversely, the linearity assumption appeared to be mostly satisfied, and there was little concern of influential observations. Furthermore, because our analysis did not rely on interpretations of individual pan-cancer coefficients, instead considering only the pooled linear predictions, these technical issues if resolved would not likely alter our conclusions. Overall, it is likely that the pan-cancer linear predictions in this study represent a combination of tumor aggressiveness and potential for response to treatment, with opportunity for optimization and increased interpretability should the above issues be addressed.

Despite these limitations, the results demonstrate that pan-cancer genomic information can improve progression risk prediction for several cancer types, although with varying efficacy. This variation reflects underlying differences between cancers and indicates that pan-cancer information should complement, rather than replace, cancer-specific analysis. Variability may relate to within-cancer genetic heterogeneity, dissimilarity of cancer-specific genomic features compared with other included cancer types, overly simplistic CoxPH model assumptions, other issues with the pan-cancer fit as outlined above, or underadjustment for variables lacking in the TCGA data set, such as comprehensive oncological treatment planning information. Although pan-cancer genomic predictions are interpretable, it is difficult to draw definitive conclusions regarding the reasons for better performance in some cancer types. Directionally consistent but statistically insignificant results in some cancer types may also simply relate to inadequate sample sizes for precise per-cancer validation.

Future work should notably focus on optimizing the prognostic information provided by the pan-cancer data, and on optimizing integration with per-cancer clinical and genomic information. The specific pan-cancer model created and presented in this paper was constructed to simply demonstrate that pan-cancer gene expression information can predict per-cancer progression; it was not optimized for predictive accuracy, and is not intended to be used as-is outside this study. Future work could optimize pan-cancer predictions via more sophisticated models, for instance regularized survival models or modern machine learning techniques. Per-cancer integration could also be optimized, for instance through the use of Bayesian methods. Per-cancer genomic predictions in this study were also flawed, and future work should investigate whether the added value of pan-cancer predictions persists when adjusted for accurate and established per-cancer indicators. Additional improvements could include multiregion sequencing and dynamic gene expression follow-up data to explore how to integrate spatiotemporal heterogeneity information, and to help incorporate complex real-world treatment plans for clinical translation of the model. These refinements could be of benefit to cancer types that did not see notable improvement from pan-cancer predictions in the current analysis.

More broadly, these findings support the use of cross-cancer genomic information to address sample size limitations that affect radiation therapy biomarker development, improving the stability of prognostic modeling. Clinical decisions after radiation therapy rely on estimates of progression risk, including decisions related to follow-up intensity, additional treatment, and clinical trial eligibility. The reliability of these estimates depends on the amount of information available to fit prognostic models. In radiation therapy studies, this information is often limited because analyses are restricted to single cancer types with modest event counts. At the same time, routine pooling of patient-level data across institutions remains difficult, limiting opportunities to increase sample size through data sharing. Pan-cancer modeling provides a practical way to increase the information used to estimate genomic effects. This study focused on radiation therapy due to our strong a priori expectation of shared genomic effects between cancer types for those patients, but the same principle should apply to any pan-cancer study population where researchers believe there should be shared genomic effects.

## Disclosures

The authors declare the following financial interests/personal relationships which may be considered as potential competing interests: Harry Glickman reports financial support was provided by Mitacs Inc. Harry Glickman reports financial support was provided by MEDTEQ+. Shirin A. Enger reports financial support was provided by Mitacs Inc. Shirin A. Enger reports financial support was provided by MEDTEQ+.

## Appendix Supplementary materials

HG_1_ARO_Revision3_SUPPLEMENT Image, application 1

HG_1_ARO_Revision3_SUPPLEMENT Image, application 1

## Footnotes

Supplementary material associated with this article can be found in the online version at doi:10.1016/j.adro.2026.102140.
