# Supplementary material for: Pan-Cancer Gene Expression Predicts Per-Cancer Radiation Therapy Response
Source: Adv Radiat Oncol. 2026 Jul 27;11(12):102140. doi: 10.1016/j.adro.2026.102140 (PMC13654517; doi:10.1016/j.adro.2026.102140)
Supplement: Supplementary file 1 [file mmc1.pdf]

Supplementary Materials

Pan-cancer gene expression predicts per-cancer radiotherapy response

September 11, 2026

Contents

1 Pan-Cancer Linear Predictions **2**

2 Per-Cancer Genomic Predictions **2**

3 Model Assumption Checks **3**

1 Pan-Cancer Linear Predictions

Linear predictions extracted from pan-cancer models for the 8 evaluation types are presented in Figure E1 as boxplots, stratified by progression status (PFI). Boxplots were created using the *ggplot2* and *ggpubr* R packages. The lower and upper hinges correspond to the first and third quartile. The upper and lower whiskers extend from the hinge to the largest and smallest values no further than 1.5 * IQR (interquartile range) from the hinge.

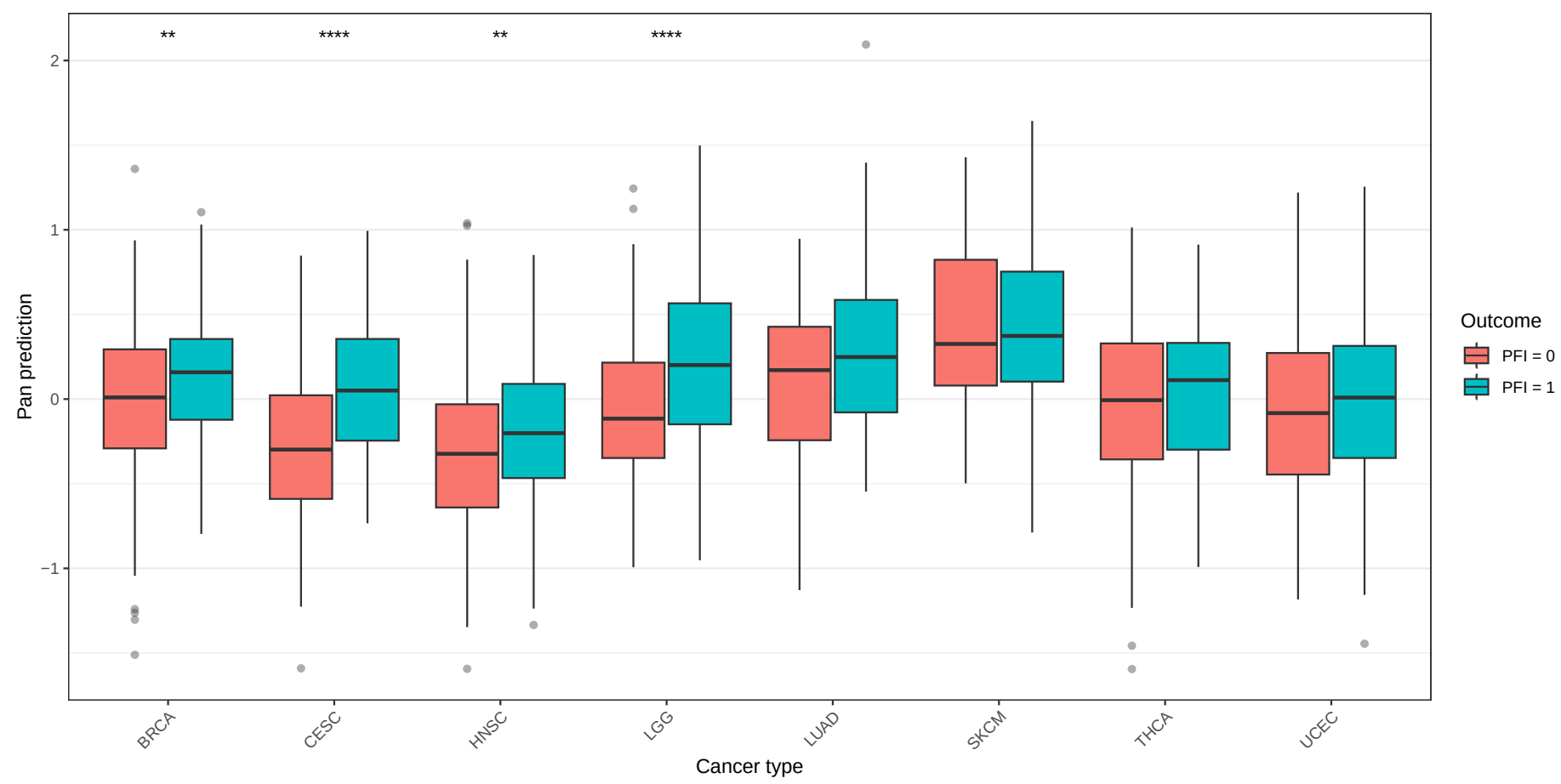

Figure E1: Linear Predictions. Linear predictions from pan-cancer models applied to per-cancer data. Boxplots are presented for each evaluation cancer type, stratified by progression status (PFI). Significance indicators are from two-sample Wilcoxon tests, *: p $\leq$ 0.05, **: p $\leq$ 0.01, ***: p $\leq$ 0.001, ****: p $\leq$ 0.0001.

2 Per-Cancer Genomic Predictions

Per-cancer genomic predictions were generated for comparison using an out-of-fold validation framework. Data for each evaluation type were split into 5 folds using the *caret* R package. Two methods were used to generate out-of-fold predictions. First, all 100 gene set parameters were used in a multivariate CoxPH model, as in the pan-cancer analysis, and linear predictions were extracted for each test case and concatenated out-of-fold. This process was repeated 10 times and concatenated predictions were averaged for stability. Second, the available TCGA sample size and the number of events were assessed to determine the maximum number of predictor parameters that could be accommodated ranging from 10 (more lenient) to 20 (more conservative) events per parameter. Dimension reduction using principal component analysis (PCA) was performed to reduce the degrees of freedom in the genomic parameters down to these ranges.

Scaling and centering preprocessing parameters were calculated based on the training folds and applied to both the training and test folds. A principal component transformation was fit on the training folds using the *prcomp* R package, and also applied to the test fold. The cumulative variance explained by chosen PCA components was averaged across sets of training folds for reporting. Multivariate CoxPH models were fit on the train fold transformed components, separately using the number of components allowed under 10 and 20 EPP rules of thumb, and linear predictions were made in the test fold. These out-of-fold linear predictions were concatenated for comparison to pan-cancer risk scores. Again, this process was repeated 10 times and out-of-fold predictions were averaged for stability.

Per-Cancer CoxPH model hazard ratios, 95% confidence intervals, and p-values for various per-cancer predictions are presented in Table E1 attached separately. Per-cancer predictions were standardized for interpretation. Models 1, 3, and 5 are progression as a function of out-of-fold per-cancer predictions generated using all 100 genomic parameters, at 10 events per parameter (EPP) using principle component analysis (PCA), and at 20 EPP using PCA, respectively. Models 2, 4, and 6 are progression as a function of the per cancer scores from models 1, 3, and 5, as well as adjustment for pan-cancer scores, age, stage, and gender. For per-cancer out-of-fold predictions, accounting for one of the five data folds being withheld, training folds had an average of 66 events for BRCA, 46 for CESC, 103 for HNSC, 117 for LGG, 71 for LUAD, 98 for SKCM, 38 for THCA, and 62 for UCEC. At a lenient 10 EPP, this results in estimated degrees of freedom for prediction of 7 for BRCA, 5 for CESC, 10 for HNSC, 12 for LGG, 7 for LUAD, 10 for SKCM, 4 for THCA, and 6 for UCEC, explaining 71%, 58%, 75%, 77%, 73%, 76%, 61%, and 68% of the variance in the train folds (in a single repeat), respectively. At a more stringent 20 EPP, this leaves 3 degrees of freedom for BRCA, 2 for CESC, 5 for HNSC, 6 for LGG, 4 for LUAD, 5 for SKCM, 2 for THCA, and 3 for UCEC, explaining 54%, 40%, 62%, 67%, 63%, 65%, 50%, and 53% of the variance in the train folds, respectively. This methodology concatenates and averages out-of-fold genomic predictions from multiple models as if they were from the same model, and therefore must be interpreted cautiously. There is no obvious workaround, however, given the impossibility of generating multivariate models that converge in single folds of small per-cancer samples. The risks of this technique are also mitigated since the model specification is the same across all sets of train folds, but future work with larger sample size should perform within-fold validation instead.

3 Model Assumption Checks

CoxPH model assumptions (proportional hazards, linearity, and influential observations) of pan-cancer and per-cancer models were tested statistically and graphically using the *cox.zph* and *ggcoxdiagnostic* functions from the *survival* and *survminer* R packages.

In pan-cancer models, there were statistical violations of the proportional

hazards assumption globally, for several genomic variables, and cancer type based on the *cox.zph* test, but with limited visually obvious patterning in schoenfeld residuals except for SKCM and OTHER cancer types. There were no obvious patterns in martingale residuals plotted against covariates, and dfbeta values were at most approximately 0.04, and much lower for most variables.

In per-cancer models, there were few statistical or visually obvious violations of the proportional hazards assumptions based on the *cox.zph* test and schoenfeld residuals, except for LUAD which had violations for age, gender, and globally. The pan-cancer score itself did not show concerning evidence of proportional hazard assumption violation in the per-cancer models. There were no obvious nonlinearities based on martingale residuals plotted against covariates. There were often relatively influential observations for stage coefficients, with dfbeta values above 0.1, but dfbeta values were always small for pan-cancer prediction coefficients.
